# Hydroxychloroquine Therapy Led to the Diagnosis of Glucose-6-Phosphate Dehydrogenase (G6PD) Deficiency in an Elderly Patient with COVID-19 Involvement: A Case Report and Review of the Literature

**DOI:** 10.1155/2022/4749424

**Published:** 2022-10-03

**Authors:** Razieh Taghizadeh-Sarvestani, Hamid Reihani, Ali Ghanei-Shahmirzadi, Alireza Keshtkar, Parsa Yazdanpanahi

**Affiliations:** ^1^Department of Pediatric Neurology, Shiraz University of Medical Sciences, Shiraz, Iran; ^2^Student Research Committee, School of Medicine, Shiraz University of Medical Sciences, Shiraz, Iran

## Abstract

Glucose-6-phosphate dehydrogenase (G6PD) deficiency is the most common RBC abnormality, affecting 400 million people globally. Neonatal jaundice, hemolytic anemia, icteric skin, dark urine, and fever are usually the primary signs of this condition, which is generally diagnosed between the ages of infancy and 16 years old. Therefore, its first manifestation in old age is an unexpected phenomenon. Here, we present the case of a 70-year-old man with no past medical history of G6PD deficiency that was admitted to our hospital due to COVID-19 infection and developed acute hemolytic anemia while receiving hydroxychloroquine (HCQ) medication for COVID-19-related pneumonia.

## 1. Introduction

Glucose-6-phosphate dehydrogenase (G6PD) deficiency is the most common RBC abnormality, affecting 400 million people globally [1, 2]. Neonatal jaundice, hemolytic anemia, icteric skin, dark urine, and fever are usually the first signs of this condition, which is generally diagnosed between the ages of infancy and 16 years old [3, 4]. Therefore, the first onset of this disease in old age is an unexpected phenomenon that is one of the rare aspects of our case. On the other hand, although G6PD is a benign hematologic condition, an acute hemolytic crisis triggered by oxidative agents such as fava beans, medications (like hydroxychloroquine (HCQ)), or infections might be its most life-threatening clinical presentation.

HCQ is a renowned drug in the treatment of malaria, and it has lately been widely used as a trial medication to treat coronavirus disease 2019 (COVID-19). HCQ is a forbidden medication in G6PD patients, but recent investigations have challenged its role in causing hemolytic anemia [5]. However, following the prevalence of COVID-19 and the widespread usage of this drug, cases of hemolytic anemia in COVID-19 patients and their recovery after discontinuation of this drug were reported, which led us to collect these studies and review them thoroughly. Here, we introduce a 70-year-old man with no past medical history of G6PD deficiency who was admitted to our hospital for COVID-19 infection and experienced acute hemolytic anemia in the setting of HCQ therapy for COVID-19-related pneumonia.

## 2. Case Presentation

**Figure 1:**
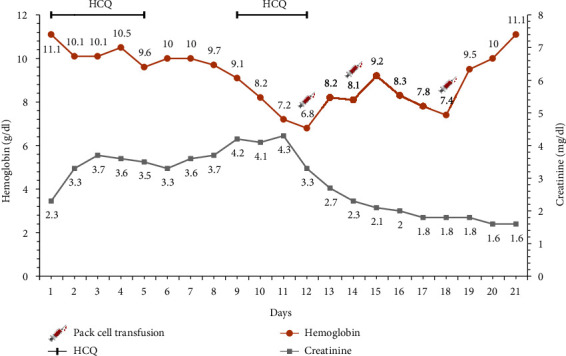
Laboratory course of the patient's hemoglobin and creatinine during hospitalization.

A 70-year-old man presented to our emergency department with a dry cough three days before admission, dyspnea, and a positive result from the recent COVID-19 PCR test. He had a medical history of diabetes mellitus type 2, high blood pressure, and hypothyroidism. After the initial evaluation, his oxygen saturation was at 90%, and a mild fever was detected. Therefore, we started with 2 liters of oxygen with a nasal cannula. A high-resolution CT (HRCT) of the chest was performed, and multiple patchy infiltrations were reported. Shortly after his admission, his oxygen saturation decreased to 75%, so we transferred him to the intensive care unit (ICU). Following our institutional protocol, he started on 800 mg HCQ on day one, then, 200 mg twice a day for four days. Then, we noticed a slight decrease in his hemoglobin level while he was receiving his first course of the HCQ, but his hemoglobin level stabilized after the first course of treatment. The laboratory courses of the patient's hemoglobin and creatinine level are illustrated in Figure 1. Afterward, a subsequent HRCT was performed and showed severe involvement of his lungs. Consequently, according to the promising results around HCQ's role in the treatment of COVID-19 at that time, we started the second treatment course with this drug; however, after three days, we noticed a severe reduction in his hemoglobin level again. Therefore, we stopped the HCQ and packed cell transfusion started for him. His peripheral blood smear (PBS) revealed peripheral schistocytes, which were in favor of hemolytic anemia. According to the previous G6PD history in his first-degree relatives, a G6PD level test was requested, and the diagnosis of G6PD was established. Ultimately, he was discharged in good condition by discontinuing HCQ as a G6PD stimulus and transfusing several bags of pack cells.

## 3. Discussion

The COVID-19 pandemic, which was initiated in December 2019, has many more dimensions yet to be revealed. One of these aspects is G6PD-deficient patients. G6PD is the most common red cell enzymatic disorder worldwide, and 400 to 500 million people are affected by one of its subtypes [1]. The role of COVID-19 in this disorder has not been adequately studied. Infections, especially viral infections by increasing oxidative stress, have previously been recognized as a trigger for G6PD deficiency, but more studies are needed on the consequences of COVID-19 in G6PD deficient patients. [6, 7]. On the other hand, the drug HCQ, an antimalarial medication, was introduced as one of the early experimental treatments for COVID-19. This drug, along with COVID-19 infection, was an additional cause to explain hemolysis in G6PD patients. Therefore, for an adequate understanding of this issue, our team conducted an advanced search strategy using keywords and mesh terms in databases such as PubMed, Scopus, Web of Science, and Google Scholar to find more cases with COVID-19 infection that used HCQ as a treatment and had hemolysis, which results are presented in Table 1.

In some cases, which we included in Table 1, HCQ administration has been recommended as the main reason for patients' hemolysis. But some of the other investigations propose otherwise. In a retrospective cohort survey by Mohammad et al. [5], in 2018, which analyzed 275 patients with rheumatic disease who had exposure to HCQ for more than 700 months, none of their 11 patients diagnosed with G6PD and simultaneously HCQ reported hemolysis was used. Similarly, in one of the cases mentioned in our table, they started HCQ on the 6th day of admission. Still, their retrospective evaluation of daily smear demonstrated a significant number of hemighost cells and microspherocytes from the 4th day of admission (2 days before they start HCQ), and their number increased gradually until the 7^th^ day [17]. Although chloroquine is mentioned in the list of drugs that can lead to hemolysis in G6PD patients [11], it does not seem that a short duration of HCQ administration, which hospitals used for the treatment of COVID-19, could cause such severe hemolysis in the absence of another oxidative stress like a systemic infection [12]. Therefore, we assume that COVID-19 infection was the principal basis and primary trigger, which in combination with HCQ as a secondary agent; it induced hemolysis in G6PD patients. The interesting point about our case is that he did not experience even one episode of a hemolytic crisis in his lifetime, despite his continuous consumption of fava beans and G6PD unmasked just after his involvement with the COVID-19 virus and HCQ administration in the hospital.

In conclusion, G6PD is the most common enzymatic disorder in red blood cells and can lead to a hemolytic crisis, which can be associated with severe consequences such as death. COVID-19 should be considered a potent oxidative stress factor in these patients, especially since it is periodically treated with G6PD-exacerbating drugs such as HCQ.

## Figures and Tables

**Table 1:** Characteristics of 11 included studies.

Author, year	Age (year), Gender, Race	Symptoms at presentation	Relevant comorbidities	G6PDhistory	Hemoglobin (g/dl)		G6PDlevel (IU/g Hg)	Peripheral blood smear	Outcome
					Baseline	crisis			
Laslett et al. 2021 [8]	60, male, African American	Fever and dizziness and mild shortness of breath	G6PD	Known	14.1	6.8	19.8	Early red cell precursors and hemighost cells	Expired (hemodynamic compromise)

Ali et al. 2021 [9]	57, male, African (Nigerian)	COVID-19 and DKA	Diabetes	New	12.4	7.4	2.8	Hemighost cells	NR

Palmer et al. 2020 [10]	62, male, Africocaribbean	Fever, dyspnea, vomiting, and diarrhea	DM type 2 and HTN	New	16	5.2	0.8	Normochromic normocytic erythrocytes and a few hemighost cells	Discharged

Obeidat et al. 2020 [11]	64, male, NR	Cough and fever	DM, hypothyroidism, and HTN	New	13.2	NR	14 (224–517)	Moderate normochromic, normocytic anemia few ovalocytes, few spherocytes, and mild rouleaux formation with mild neutrophilic leukocytosis with mild absolute lymphopenia	NR

Mastroianni et al. 2020 [12]	32,male, sub-Saharan African	NR	Nothing	New	10	7.7	Below 0.2	No schistocytes or reticulocytosis	Discharged

Maillart et al. 2020 [13]	65, male, African	Hypoxemia	HTN and type 2 diabetes	New	13.3	Below detection	Below 0.2	NR	NR

Kuipers et al. 2020 [14]	56, male, NR	Myalgia and a dry cough	DM type2	New	11.4	NR	0.1	Blister cells	NR

Dickinson et al. 2020 [15]	60, male, African American	Fever and shortness of breath	HTN and type 2 diabetes	New	12	6.8	Normal	Schistocytes and spherocytes	Expired (refractory shock)

Franceschi et al. 2020 [16]	72, male, Caucasian	Fatigue, dyspnea, dizziness, and fever	Ischemic cardiomyopathy	New	15	12.5	NR	Anisopoikilocytosis; reticulocytes as large and round-shaped cells; some “hemighost”	NR

Beauverd et al. 2020 [17]	68, male, Congolese	Fever, dyspnea, muscular pain, and tiredness	Type 2 DM, HTN, and chronic renal insufficiency	New	12	6.5	2.5	Numerous hemi-ghost cells and microspherocytes	NR

Aguilar et al. 2020 [18]	51, male, African American	Fevers, myalgia, and dry cough, and worsening shortness of breath	Type 2 DM, HTN, and morbid obesity	New	14.5	5.9	Abnormal	Positive schistocyte	Discharged

NR, not reported; DKA, diabetic ketoacidosis; G6PD, glucose-6-phosphate dehydrogenase; DM, diabetes mellitus; HTN, hypertension.

## Data Availability

The data used to support the findings of this study are available from the corresponding author upon reasonable request.
